# Trends in the Incidence of Cervical Cancer in Jordan, 2000–2013

**DOI:** 10.1155/2017/6827384

**Published:** 2017-08-27

**Authors:** Ghazi Sharkas, Kamal Arqoub, Yousef Khader, Omar Nimri, Wejdan Shroukh, Hala Jadallah, Tayseer Saheb

**Affiliations:** ^1^Field Epidemiology Training Program, Non-Communicable Diseases, Ministry of Health, Amman, Jordan; ^2^Jordan Cancer Registry, Ministry of Health, Amman, Jordan; ^3^Department of Public Health, Jordan University of Science & Technology, Irbid, Jordan; ^4^Jordan University, Amman, Jordan; ^5^Department of Nutrition, Royal Medical Services, Amman, Jordan; ^6^Department of Family Medicine, Ministry of Health, Amman, Jordan

## Abstract

**Objectives:**

To determine the incidence of cervical cancer in Jordan and assess its trend in over a 14-year period (2000–2013).

**Methods:**

This descriptive study was based on secondary analysis of cervical cancer data that are registered in the Jordan Cancer Registry (JCR).

**Results:**

A total of 591 women were diagnosed with cervical cancer in Jordan during the period 2000–2013. The age at diagnosis ranged between 15 and 97 years, with a median of 50 years. The average age standardized rate (ASR) was 2.0/100,000 women. The incidence of cervical cancer started to decrease after 2006 but it remained relatively constant between 2008 and 2013. Over the 14-year period, ASR for cervical cancer decreased by 28.6% from 2.1 per 100,000 women in 2000 to 1.5 per 100,000 women in 2013. About 46.5% of the cases were of squamous cell carcinoma morphology. Early cancer constituted about 60% of the cases, regional cases constituted 9.6%, and distant metastatic cases constituted 10.7%.

**Conclusions:**

The incidence of cervical cancer in Jordan is low compared to regional estimates and remained relatively constant between 2008 and 2013. Implementation of screening measures could lead to better case finding, early diagnosis, and prevention of cervical cancer.

## 1. Introduction

Worldwide, cervical cancer is the fourth most common cancer in women. In 2012, according to the GLOBOCAN estimates [1], there were 572,624 new cases of cervical cancer and there were an estimated 265,672 deaths from cervical cancer worldwide accounting for 7.5% of all female cancer deaths. Mortality from cervical cancer varies 18-fold between the different regions of the world with about 87% of cervical cancer deaths occurring in the less developed regions [2].

In 2012, the cumulative risk of cervical cancer in women aged below 75 years was 1.42% worldwide [1]. In developing countries, cervical cancer is the second most common cancer and the third leading cause of cancer death [1]. In the Middle East, the incidence of cervical cancer is lower than that in developed countries with most cases of cervical cancer detected at a late stage [3].

The trend of cervical cancer depends on the availability of effective screening programs and time changes in the profile of risk factors [4–6]. The incidence of invasive cervical cancer has decreased in developed countries in a steady manner over the last few decades. Over the last 30 years, there have been significant decline in the incidence of invasive cervical cancer in many developed countries [7] including USA [8] and Canada [9, 10]. However, the reductions in the incidence of cervical cancer varied according to race and histology of cervical cancer. In a study [6] that assessed trends in cervical cancer across 38 countries in five continents, strong decreasing trends in cervical cancer risk were seen in the highest-income countries, while no obvious changes were found in lower-income countries. In developing countries, the incidence of cervical cancer has changed a little, except for countries that have witnessed demographic and epidemiologic transition [5, 11]. The geographic variation in cervical cancer rates are due to differences in the availability of screening, which can prevent the development of cancer through the detection and removal of precancerous lesions, and the prevalence of human papillomavirus (HPV) infection [12–14].

Although the epidemiology of cervical cancer is well known in many countries of the world, there are scarcity of data in the Eastern Mediterranean region and Arab countries including Jordan [10]. In Jordan, there is no established national screening program for cervical cancer. Nevertheless there are scattered efforts of screening in the Ministry of Health, Jordan Association for Family planning (JAFP), and the private sector. The screening services are not institutionalized yet, and no database is available. This study was conducted to determine the incidence of cervical cancer in Jordan and assess trend in incidence of cervical cancer in Jordan over a 14-year period (2000–2013).

## 2. Methods

This descriptive study was based on secondary analysis of cervical cancer data that are registered in the Jordan Cancer Registry (JCR). The study utilized all data about cervical cancer cases among Jordanians that were registered in the JCR during the period from 2000 to 2013. Cases of cervical cancer for non-Jordanian patients were excluded from this study. The cancer cases were collected actively by JCR staff and passively by trained focal points in all hospitals in Jordan including governmental, military, private, and university hospitals, as well as main clinics and laboratories. In JCR, the trained registry staff collect the data about clinical and/or histopathological diagnosis through regular field visits and through the trained focal points who report cancer cases to JCR through filling a special form. After obtaining the official permission from Jordan Ministry of Health through the standard data request form, the data were obtained and retrieved from the JCR database. The data included information on patients' name, national number, sex, age, address, telephone number, and nationality as well as information on tumor variables (date of diagnosis, primary site, histopathology, behavior, grade, stage, and basis of diagnosis). Duplicate entries were identified and corrected by checking the national number, patient's name and age, diagnosis, and patient residency place.

All incident cases of cervical cancer were identified mostly on the basis of histopathological reports. The validity of data was verified through internal quality checks, external checks, and computer checks. Cancer data were classified by the primary site (topography) and histopathology (morphology) and then coded according to the International Classification of Diseases for Oncology 3rd edition (ICDO-3) published by WHO in the year 2000.

Ethical approval to conduct the study was obtained by the Ethical Committee at Jordan University of Science and Technology. Annual crude and age standardized incidence (ASR) rates were calculated. World standard population was used for standardization of rates for national and international comparisons and expressed as cases per 100,000 women. Data entry and analysis were done using Can-Reg 4.31 software developed by the International Agency for Research on Cancer (IARC), Lyon, France. Excel sheet was used to develop the figure that shows the trend of cervical cancer over time.

## 3. Results

A total of 886 women were diagnosed with cervical cancer in Jordan during the period from 2000 to 2013. Of those, 591 (66.7%) were Jordanians. Only Jordanian women with cervical cancer were included and analyzed in this study. The age of Jordanian women at diagnosis with cervical cancer ranged between 15 and 97 years, with an overall median of 50 years. Of all cases, 79.4% were diagnosed in the middle region, 17.9% in northern region, and 2.7% in the southern region. The majority of patients (95.7%) were ever married.

The average annual incidence rate was 1.5/100,000 women population.  Table 1 shows the total number of cervical cancer cases in the period 2000–2013 and average annual incidence rate according to the age group. The incidence rate was the highest in women aged 85 plus years (12.7/100,000), followed by women in the age group of 70–74 years (11.4/100,000).

**Figure 1:**
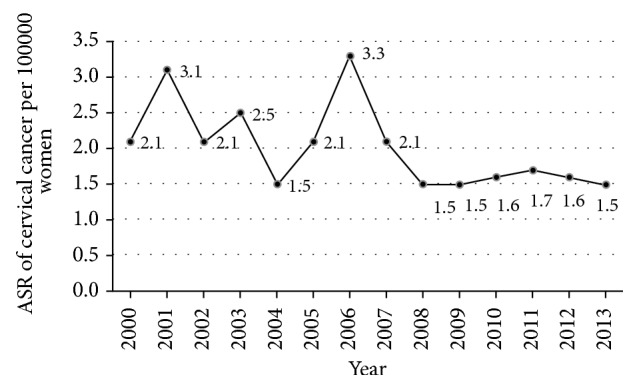
Trends in the age standardized rate (ASR) of cervical cancer between 2000 and 2013.

The average ASR was 2.0/100,000 women. The ASR fluctuated between 2000 and 2007 with the highest ASR seen in 2001 and 2006. It seems that the incidence of cervical cancer started to decrease after 2006 but it remained relatively constant between 2008 and 2013. Over the 14-year period, ASR for cervical cancer decreased by 28.6% from 2.1 per 100,000 women in 2000 to 1.5 per 100,000 women population in 2013 (Figure 1).

The distribution of cervical cancers by the tumor morphology is shown in Table 2. About 46.5% of the cases were of squamous cell carcinoma morphology and 14.4% were of adenocarcinoma morphology. According to stage, early cancer constituted about 60% of the cases (8.8% in situ and 43.1% localized), regional cases constituted 9.6%, and distant metastatic cases constituted 10.7%. Cases with unknown stage constituted 27.7%.

## 4. Discussion

Cervical cancer incidence is generally low in the Middle East and does not appear to be increasing [1, 3]. Among women in Jordan, cervical cancer ranks as the 15th most common cancer. Furthermore, it ranks as the 10th most common cancer among women aged between 15 and 44 years [15]. This study showed that the average ASR over the period between 2000 and 2013 was 2.0 per 100,000 women. This rate is lower than the worldwide incidence rate and lower than that in developed countries [1, 2], even though women in Jordan have less access to cervical cancer screening and prevention programs. The low incidence may be a result of underreporting and inadequate case findings. Moreover, this may be due to societal disapproval of extramarital sexual activity in Jordan. However, data on the burden of Human Papilloma Virus (HPV) infection in Jordan are scarce. Therefore, there is a need for more studies to map out the HPV infection load in the female population of Jordan.

Compared to other countries in the region, Jordan has the lowest rate of cervical cancer [1]. Compared to the incidence rate in Jordan, the GLOBOCAN 2012 estimates (per 100,000 women population) [1] showed higher rates in the Eastern Mediterranean countries including United Arab Emirates (9.8), Bahrain (5.9), Oman (5.3), Qatar (5.1), Lebanon (4.6), Israel (4.6), Turkey (4.3), Kuwait (4.0), Yemen (3.1), Iraq (2.8), Saudi Arabia (2.7), Syria (2.6), and Palestine (2.0).

Moreover, the Middle East Cancer Consortium (MECC) data on cervical cancer in populations in Cyprus, Egypt, Israel, and Jordan for the period 1996–2001 [3] showed that the highest ASR of cervical cancer was in Israeli Jews (5.3), followed by Cypriots (3.7), Egyptians (2.7), Jordanians (2.6), and Israeli Arabs (2.5). The most recent published study on the incidence and trend of cervical cancer in the Eastern Mediterranean region was conducted in Iran and showed that the incidence rate is low but trend is increasing [16]. In 2013, the incidence rates of cervical cancer were the lowest in Australasia, North Africa, Middle East, and high-income North America and the highest in Oceania, eastern sub-Saharan Africa, and western sub-Saharan Africa [17].

The variations in the incidence rates reflect the differences in sexual activity. Clearly the HPV is the prime risk factor and the Muslim religious background is naturally of great significance in this regard. The implementation of successful cervical cancer screening programs in most developed countries may, in the short term, reveal more cases but would not in the long term account for a higher incidence [4].

Screening programs are not in place in Jordan as well as most countries in the region. Of the 98 physicians who participated in a study in the UAE, only 40% reported ever having performed a Pap smear. Therefore, training programs on cervical screening are deemed to be very necessary [18]. In Jordan, little is known about Pap smear services in Jordan. The 2007 Behavioral Risk Factor survey [19] showed that only 28% of married women had reported having a papanicolaou test, while about 75% had never heard of pap smear. Jordanian women who had a Pap smear had it on opportunistic basis and clearly prefer a female doctor to perform the swab. Another study showed that about one-third of women were unaware of the significance of a positive cervical smear and three-quarters did not know the causes of neoplastic development [20].

Furthermore, there may be some underreporting in the countries of the region including Jordan. In a prospective study in Saudi Arabia the percentage of abnormal Pap smears was 4.7%, much higher than the 1.6% reported in the compounded literature [21].

In conclusion, the incidence of cervical cancer in Jordan during the study period was considered very low compared to regional and international incidence rates. The incidence of cervical cancer started to decrease after 2006 but it remained relatively constant between 2008 and 2013. Implementation of screening measures including Pap smears which are currently applied in developed countries could lead to better case finding, early diagnosis, and prevention of cervical cancer.

## Figures and Tables

**Table 1:** The total number of cervical cancer cases in the period 2000–2013 and average annual incidence rate according to the age group.

Age (year)	Number of cases	Average annual incidence rate per 100,000 women population
15–19	2	0.1
20–24	1	0.1
25–29	16	0.5
30–34	32	1.1
35–39	61	2.5
40–44	79	4.3
45–49	96	7.4
50–54	66	6.7
55–59	63	7.0
60–64	61	8.4
65–69	47	8.8
70–74	41	11.4
75–79	9	5.0
80–84	6	5.1
85+	9	12.7

**Table 2:** Distribution of patients with cervical cancer according to morphology in Jordan, 2000–2013.

Morphology	*N*	%
Squamous cell carcinoma	275	46.5
Keratinized squamous cell carcinoma	38	6.4
Non-keratinized squamous cell carcinoma	67	11.3
Squamous intraepithelial neoplasia	14	2.4
Adenocarcinoma	85	14.4
Carcinoma	32	5.4
Adenosquamous carcinoma	15	2.5
Malignant tumor	9	1.5
Endometrioid carcinoma	9	1.5
Other types	47	8.0
